# Association of Parkinson’s Disease and Its Subtypes with Agricultural Pesticide Exposures in Men: A Case–Control Study in France

**DOI:** 10.1289/ehp.1307970

**Published:** 2015-03-27

**Authors:** Frédéric Moisan, Johan Spinosi, Laurène Delabre, Véronique Gourlet, Jean-Louis Mazurie, Isabelle Bénatru, Marcel Goldberg, Marc G. Weisskopf, Ellen Imbernon, Christophe Tzourio, Alexis Elbaz

**Affiliations:** 1 Département santé travail, Institut de veille sanitaire, Saint-Maurice, France; 2 INSERM, U708, Neuroepidemiology, Bordeaux/Paris, France; 3 Unité mixte de recherche épidémiologique et de surveillance en transport, travail et environnement (Umrestte), Université Lyon, Lyon, France; 4 Caisse départementale de la Gironde, Mutualité sociale agricole, Bordeaux, France; 5 Service de Neurologie, CHU Poitiers, Poitiers, France; 6 Université Versailles St-Quentin, UMRS 1018, Villejuif, France; 7 Department of Environmental Health, Harvard T.H. Chan School of Public Health, Boston, Massachusetts, USA; 8 INSERM U1018, Centre for Research in Epidemiology and Population Health, Villejuif, France

## Abstract

**Background:**

Pesticides have been associated with Parkinson’s disease (PD), but there are few data on important exposure characteristics such as dose–effect relations. It is unknown whether associations depend on clinical PD subtypes.

**Objectives:**

We examined quantitative aspects of occupational pesticide exposure associated with PD and investigated whether associations were similar across PD subtypes.

**Methods:**

As part of a French population-based case–control study including men enrolled in the health insurance plan for farmers and agricultural workers, cases with clinically confirmed PD were identified through antiparkinsonian drug claims. Two controls were matched to each case. Using a comprehensive occupational questionnaire, we computed indicators for different dimensions of exposure (duration, cumulative exposure, intensity). We used conditional logistic regression to compute odds ratios (ORs) and 95% confidence intervals (CIs) among exposed male farmers (133 cases, 298 controls). We examined the relation between pesticides and PD subtypes (tremor dominant/non-tremor dominant) using polytomous logistic regression.

**Results:**

There appeared to be a stronger association with intensity than duration of pesticide exposure based on separate models, as well as a synergistic interaction between duration and intensity (*p-*interaction = 0.04). High-intensity exposure to insecticides was positively associated with PD among those with low-intensity exposure to fungicides and vice versa, suggesting independent effects. Pesticide exposure in farms that specialized in vineyards was associated with PD (OR = 2.56; 95% CI: 1.31, 4.98). The association with intensity of pesticide use was stronger, although not significantly (*p*-heterogeneity = 0.60), for tremor-dominant (*p-*trend < 0.01) than for non-tremor–dominant PD (*p-*trend = 0.24).

**Conclusions:**

This study helps to better characterize different aspects of pesticide exposure associated with PD, and shows a significant association of pesticides with tremor-dominant PD in men, the most typical PD presentation.

**Citation:**

Moisan F, Spinosi J, Delabre L, Gourlet V, Mazurie JL, Bénatru I, Goldberg M, Weisskopf MG, Imbernon E, Tzourio C, Elbaz A. 2015. Association of Parkinson’s disease and its subtypes with agricultural pesticide exposures in men: a case–control study in France. Environ Health Perspect 123:1123–1129; http://dx.doi.org/10.1289/ehp.1307970

## Introduction

Previous studies have shown an association between Parkinson’s disease (PD) and exposure to pesticides. A recent meta-analysis of 39 studies reported a 60% increased risk of PD associated with exposure to pesticides (van der Mark et al. 2012); analyses based on a smaller number of studies (insecticides, *n* = 14; herbicides, *n* = 14; fungicides, *n* = 9) showed this association to be mainly explained by insecticides and herbicides. This meta-analysis highlighted that few studies collected detailed pesticide exposure data, and many uncertainties remain on important exposure characteristics. In particular, few studies assessed dose–effect relations, and there is little information on quantitative aspects of exposure, such as duration or intensity of exposure.

PD is a heterogeneous phenotype with different clinical subtypes (i.e., young-onset PD, tremor/non-tremor dominant) that have been defined based on key clinical features (Selikhova et al. 2009; van Rooden et al. 2010). There is some evidence that these subtypes differ in terms of pathophysiological mechanisms or disease progression (Eggers et al. 2012; Selikhova et al. 2009), and it has been suggested that different causative factors may be involved in patients with different clinical subtypes (Marras and Lang 2013; Obeso et al. 2010). However, it has not been explored whether pesticides are differentially related to different PD subtypes.

As part of a French case–control study conducted among a highly exposed agricultural population, we sought to determine characteristics of pesticide use associated with PD (dose–effect relations, broad classes of pesticides, type of agriculture), and to investigate whether the association between pesticides and PD was similar across its clinical subtypes.

## Methods

*Participants.* A population-based case–control study was conducted in five French districts (départements: Charente-Maritime, Côte-d’Or, Gironde, Haute-Vienne, and Mayenne) among members of the Mutualité Sociale Agricole (MSA), the only health insurance for farmers and workers in agriculture. The Ethical Committee of the Pitié-Salpêtrière University Hospital approved the study protocol; all participants gave informed consent. Because pesticides are applied mostly by men, the present analyses were restricted to men who were farmers and reported exposure to pesticides. However, the present study population is a subset of a larger study population that also included women and people without pesticide exposures.

We used MSA computerized databases (2006–2007) to identify district residents who bought at least one antiparkinsonian drug (code N04 of the Anatomical Therapeutic Chemical Classification System) and/or who received free medical care for PD (Moisan et al. 2011); in France, PD belongs to a list of 30 long-standing illnesses for which free medical care is granted, usually after a neurologist confirms the diagnosis. We excluded PD patients ≥ 80 years of age, those with disease duration > 15 years (information available for those with free medical care), and those receiving free medical care for dementia or psychiatric conditions. Persons who used levodopa, entacapone, tolcapone, ropinirole, pramipexole, apomorphine, bromocriptine, or selegiline were directly contacted by phone and invited to be examined by a neurologist unless *a*) they reported taking small doses of dopamine agonists for restless leg syndrome, *b*) treatment was discontinued after ≤ 1 month, or *c*) there was a documented history of drug-induced parkinsonism. Persons who used only piribedil, amantadine, or anticholinergic agents (i.e., drugs rarely used for PD) were first contacted by mail; they were asked why these drugs had been prescribed, and those who answered PD/parkinsonism or did not know were invited to be examined by a neurologist. We did not contact women ≤ 50 years of age using small doses of bromocriptine for short periods (lactation suppression) or persons using anticholinergic agents with neuroleptics (drug-induced parkinsonism). One neurologist per district examined persons who agreed to participate and used standardized criteria to diagnose PD (Bower et al. 1999). Neurologists collected clinical information (age at PD onset, symptoms at PD onset, symptoms at the time of clinical examination, and symptom improvement by treatment).

Controls were randomly selected among all MSA members who did not use antiparkinsonian drugs or receive free medical care for PD, dementia, or psychiatric conditions. For each case, we randomly selected 10 controls of similar age (within 2 years), sex, and district of residence and contacted them until 2 agreed to participate in the study. Potential controls were contacted by telephone, and those who confirmed that they did not have PD or tremor were invited to participate.

*Clinical subtypes.* Based on information collected by the neurologists, we classified PD cases into four subtypes (Selikhova et al. 2009): “early disease onset” (age at onset, < 55 years); “tremor dominant” (rest tremor as the main initial symptom, and/or dominance of tremor over bradykinesia and rigidity according to the neurologist); “non-tremor dominant” (bradykinesia and/or rigidity as main initial symptoms, and/or dominance of bradykinesia and/or rigidity over tremor according to the neurologist); and “unknown subtype” for patients that we could not classify into any of these groups, mainly due to missing values of the relevant variables. Because there was only one medical examination, we were not able to assess the rate of disease progression nor were we able to identify cases with the “rapid disease progression and old age at onset” subtype.

*Exposure assessment.* Information on education, place of residence, smoking (ever smoking; start/end years), coffee drinking (ever drinking; start/end years), family history of PD (parents/siblings), and cognitive function [Mini-Mental State Examination (MMSE)] were obtained during in-person interviews.

Participants provided detailed information on their occupational history (all occupations held ≥ 6 months since the age of 12 years) through a self-administered questionnaire. Each occupation was coded by an industrial hygienist (L.D.) blinded to disease status using the *International Standard Classification of Occupations* (International Labour Organization 1968). We classified participants as farmers if their longest occupation was “6-0: Farm Managers and Supervisors,” “6-1: Farmers,” or “6-2: Agricultural and Animal Husbandry Workers.” Use of pesticides for gardening was assessed through self-report.

For participants working in agriculture (i.e., potentially exposed to pesticides), we used a specific face-to-face questionnaire to obtain a list of all farms/firms where they had worked and a detailed description of each farm/firm, including crops (type, surface), livestock (type, number), and other activities (e.g., spraying of seeds, weeding). For each activity, we asked whether participants had personally sprayed pesticides, and we obtained relevant information: years of spraying (start/end), average annual frequency of spraying, the surface treated, and class (herbicides, fungicides, insecticides). We did not ask systematic questions about specific chemicals, but participants were invited to provide names of products if they remembered them. Questionnaires were reviewed by an industrial hygienist (L.D.), an agricultural engineer (J.S.), an agronomist (F.M.), and an epidemiologist (A.E.) blinded to disease status to verify data plausibility and consistency. For 18% of questionnaires, we contacted the participants to check their data and ask specific question raised by the experts.

Because there is evidence that neuronal loss in the substantia nigra has already started in the 5 years preceding the onset of motor symptoms (Savica et al. 2010), only exposures occurring ≥ 5 years before the reference date were considered in the analyses; the reference date was the year of PD onset in cases and the same year in matched controls (see Supplemental Material, Figure S1). We computed three indicators characterizing different dimensions of exposure: duration (the cumulative number of years with at least one application of pesticides); cumulative exposure (the lifetime number of applications); and average intensity (the average of annual frequency of applications). These indicators were computed for pesticides overall and for each class of pesticides (insecticides, fungicides, herbicides).

Participants may encounter difficulties in recalling what specific pesticides had been used in the past. An alternative approach is to indirectly assess exposure to pesticides based on types of farming because pesticide use patterns (i.e., products, spraying frequency/duration, quantity, and equipment) strongly depend on farming types, which are considerably easier to remember than pesticides. We defined the types of farming following the same approach as the European commission (Commission of the European Communities 1996). Briefly, for each type of production, we calculated the economic profit (standard gross margin) by multiplying the number of hectares (for crops) or heads (for livestock) by the reference profit for the corresponding production (Eurostat 2010). Each farm was then classified into 1 of 16 types of farming according to the ratio of each production’s profit to the farm’s total profit (Farm Accountancy Data Network 2009a). This allows classifying farms according to their main production, which is likely to represent the main source of pesticide exposure.

*Statistical analyses.* Analyses were performed using SAS 9.2 (SAS Institute Inc., Cary, NC, USA) and Stata 10.1 (StataCorp, College Station, TX, USA). *p*-Values were two-sided, and the significance level was set at 0.05.

PD and exposure to pesticides. We used conditional logistic regression to compare characteristics of PD cases and matched controls and to estimate odds ratios (ORs) and 95% confidence intervals (CIs). We performed analyses for pesticides overall and for three broad classes of pesticides (insecticides, fungicides, herbicides). We categorized indicators of exposure (duration, cumulative, intensity) into four levels according to quartiles of their distribution among exposed controls; the lowest level represented the reference group. For classes of pesticides, unexposed participants and those in the first quartile of exposure were combined to form the reference group. We used the categories’ medians to test for trend (Greenland 1995). We examined interactions between exposure indicators by including multiplicative terms. For these analyses, we dichotomized indicators at their median for overall pesticide exposure. For classes of pesticides, the three highest quartiles were combined and compared with unexposed participants and those in the first quartile. Akaike information criterion (AIC) allowed comparison of models that included different exposure indicators; lower AIC values indicate better fit.

Farmers and non-farmers differed on many characteristics, including age, smoking, MMSE score, education, and place of residence. To limit residual confounding, ORs for the association between PD and pesticide use were estimated among exposed male farmers.

Analyses were performed using conditional regression to account for matching on age and district, and all models were additionally adjusted for smoking (never, < 25 years, ≥ 25 years), coffee drinking (never, < 51 years, ≥ 51 years), tertiles of MMSE score (< 26, 26–28, > 28), family history of PD (yes/no), and use of pesticides for seeds (ever/never).

PD and type of farming. For types of farming with > 10 exposed men, we defined dichotomous variables (ever/never used pesticides for a given type) and ordinal variables (never, < median, ≥ median exposure among controls exposed to pesticides for a given type). All participants included in these analyses were exposed to pesticides, and analyses were adjusted for the same covariates as described above.

Clinical subtypes of PD and exposure to pesticides. To investigate the relation between pesticides and the two main clinical subtypes of PD among men, we used polytomous logistic regression with a three-level outcome variable [controls (reference), tremor dominant, non-tremor dominant]; this approach allowed us to perform a test of heterogeneity of the association of pesticides with the two subtypes (Morris et al. 2010). Cases with young-onset PD were excluded from these analyses due to small numbers (*n* = 22). Because these analyses involved breaking the matching, they were adjusted for matching variables (5-year age groups, district of residence). For classes of pesticides, indicators of exposure were categorized according to tertiles.

Sensitivity analyses. We excluded exposures occurring 2, 10, or 20 years before the reference date. We examined whether using two alternative definitions of being a farmer or agricultural worker affected our findings: Participants were classified as “farmers” *a*) if they had worked at least once as “farm managers and supervisors,” “farmers,” or “agricultural and animal husbandry workers”; or *b*) if they had always held one of these occupations. We investigated whether adjusting our analyses for use of pesticides for gardening (ever/never) had an influence. To assess incidence–prevalence bias, we restricted our analyses to cases with short disease duration (< 3 years) and matched controls.

## Results

**Figure 1:**
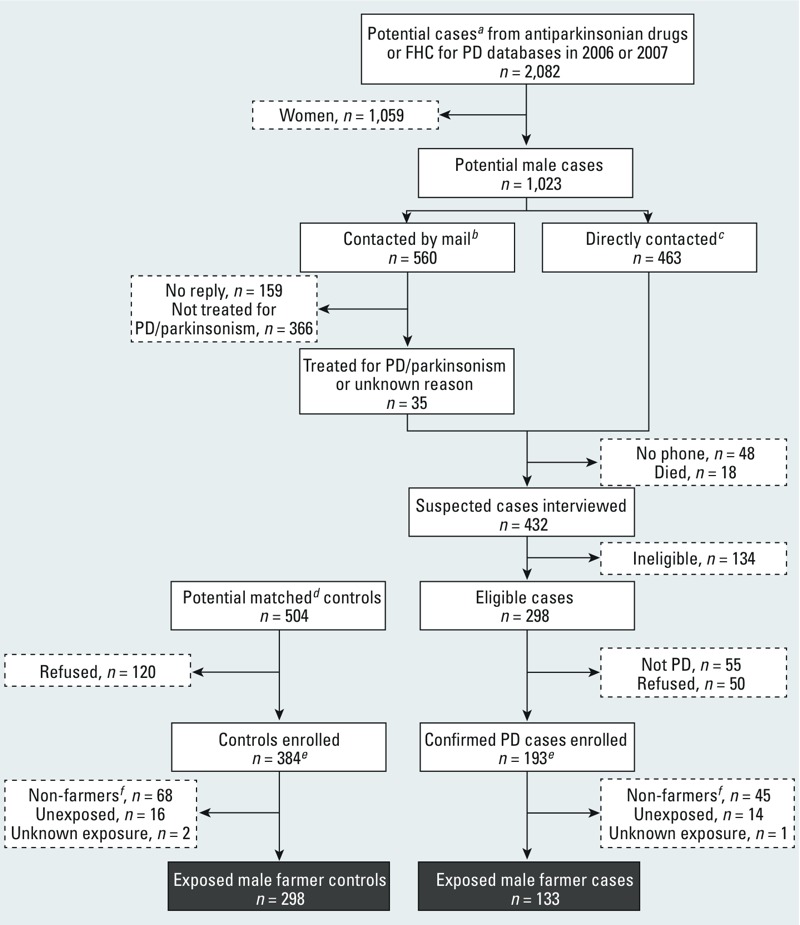
Flowchart for the inclusion of male farmer cases and controls in the study. ***^a^***Persons < 80 years of age and without free health care (FHC) for dementia or psychiatric conditions. ***^b^***Persons with at least one prescription of piribedil, amantadine, or anticholinergics who did not use other antiparkinsonian drugs. ***^c^***Persons with at least one delivery of levodopa, entacapone, tolcapone, ropinirole, pramipexole, apomorphine, bromocriptine, or selegiline. ***^d^***Matching for age (± 2 years), sex, and district. ***^e^***191 triplets (1 case and 2 controls) and 2 pairs (1 case and 1 control). ***^f^***Non-farmers included workers in food and beverage processors; clerical supervisors, including bookkeepers, cashiers, and related workers; and life scientists and related technicians.

*Participant’s characteristics.* Among men who verified inclusion criteria and were invited to participate as cases (*n* = 298), 248 (83%) accepted, of whom 193 had PD; they were matched to 384 controls (acceptance rate, 76%) (Figure 1). Of these, 148 (77%) cases and 316 (82%) controls were farmers; 15 cases (10%) and 18 controls (6%) had no/unknown exposure to any pesticide and represented a selected group (e.g., managers, supervisors, hired workers) or had worse cognitive function, which is likely to have affected recall (median MMSE score = 22.5 for participants with no/unknown exposure compared with 26.0 for exposed participants). Analyses are therefore based on exposed male farmers (133 cases, 298 controls). Cases were less often smokers and less likely to drink coffee than controls (Table 1).

**Table 1:** Characteristics of male cases and controls.

Characteristic	Cases	Controls
*n*	133	298
Age at study (years) in 2007, median (25th–75th percentile)	75 (70–77)	75 (70–78)
District, *n* (%)
Charente-Maritime	34 (26)	76 (26)
Côte-d’Or	17 (13)	42 (14)
Gironde	27 (20)	49 (16)
Haute-Vienne	20 (15)	47 (16)
Mayenne	35 (26)	84 (28)
Ever smoking, *n* (%)	45 (34)	154 (52)
Duration (years), median (25th–75th percentile)	22 (9–33)	30 (17–42)
Ever coffee drinking, *n* (%)	102 (77)	250 (84)
Duration (years), median (25th–75th percentile)	50 (43–55)	54 (45–60)
MMSE, median (25th–75th percentile)	26 (22–28)	27 (24–28)
Pesticide use for gardening, *n* (%)	90 (68)	192 (64)
Family history of PD (parents/siblings), *n* (%)	14 (11)	10 (3)
Parents (at least one) worked as farmers, *n* (%)	125 (94)	277 (93)
Education (completed high school and higher), *n* (%)	5 (4)	11 (4)
Rural living,^*a*^ *n* (%)	130 (98)	290 (97)
Abbreviations: MMSE, Mini-Mental State Examination; PD, Parkinson’s disease. ^***a***^Based on the longest place of residence.		

*PD and exposure to pesticides.* Cases and controls usually started applying pesticides as teenagers (median age, 17 years) and used pesticides for a median of 39 years; herbicides were the most frequently used class (88% cases, 88% controls), followed by insecticides (87% cases, 86% controls) and fungicides (80% cases, 77% controls). Pesticides were used primarily for crops (90% of applications).

**Figure 2:**
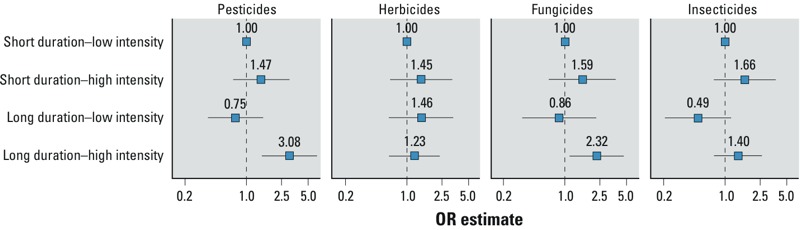
Interaction between duration and intensity of exposure to pesticides, herbicides, fungicides, and insecticides for the risk of PD among male farmers. Duration and intensity of exposure to pesticides were dichotomized according to the median of their distribution. For classes (herbicides, fungicides, insecticides), short duration or low intensity were represented by the first quartile, and long duration or high intensity by grouping the second, third, and fourth quartiles (Table 3). ORs are adjusted for age, district, duration of smoking, duration of coffee drinking, MMSE, family history of PD, and use of pesticides for seeds. Error bars are 95% CIs. *p*-Values for the interaction between duration and intensity are 0.04 for pesticides, 0.31 for herbicides, 0.38 for fungicides, and 0.34 for insecticides. Abbreviations: MMSE, Mini-Mental State Examination; PD, Parkinson’s disease.

Table 2 shows the results of associations with pesticides overall. Compared with participants in the lowest quartile, those in the highest quartile of cumulative number of applications (OR = 2.31; 95% CI: 1.09, 4.90; *p*-trend = 0.01) and average number of applications per year (OR = 2.68; 95% CI: 1.21, 5.93; *p*-trend = 0.04) had an increased PD risk. No association was observed with duration. The model including average exposure intensity had the lowest AIC value. A significant interaction (*p*-interaction = 0.04; Figure 2) was observed between duration and intensity (AIC = 353.2). Compared with male farmers with short-duration–low-intensity exposure, those with long-duration–high-intensity had the highest risk (OR = 3.08; 95% CI: 1.51, 6.27). The corresponding OR for short-duration–high intensity exposure was > 1 (OR = 1.47; 95% CI: 0.72, 3.00), while the OR for long duration–low intensity was < 1 (OR = 0.75; 95% CI: 0.37, 1.53).

**Table 2:** Association of PD with indicators of professional exposure to pesticides among male farmers.

Model	Quartile (min–max)	Cases (*n* =133) *n* (%)	Controls (*n* = 298) *n* (%)	OR (95% CI)^*a*^	*p*-Trend	AIC
Duration of exposure (cumulative number of years of exposure)						
	1 (4–29)	29 (22)	75 (25)	Reference
	2 (30–37)	30 (22)	87 (29)	0.91 (0.45, 1.81)
	3 (38–43)	41 (31)	63 (21)	1.60 (0.82, 3.14)
	4 (> 43)	33 (25)	73 (25)	1.16 (0.59, 2.27)	0.385	363.8
Cumulative exposure (cumulative number of applications)^*b*^						
	1 (4–108)	26 (20)	73 (25)	Reference
	2 (109–210)	29 (22)	73 (25)	0.92 (0.45, 1.87)
	3 (211–460)	34 (26)	73 (25)	1.34 (0.67, 2.67)
	4 (> 460)	43 (32)	73 (25)	2.31 (1.09, 4.90)	0.013	360.2
Average exposure intensity (average number of applications/year)^*b*^						
	1 (0.50–3.20)	23 (17)	73 (25)	Reference
	2 (3.22–6.01)	28 (21)	73 (25)	1.12 (0.55, 2.28)
	3 (6.04–12.51)	40 (30)	73 (25)	2.30 (1.12, 4.71)
	4 (> 12.51)	41 (31)	73 (25)	2.68 (1.21, 5.93)	0.043	358.6
Abbreviations: AIC, Akaike information criterion; max, maximum; min, minimum; MMSE, Mini-Mental State Examination; PD, Parkinson’s disease. ^***a***^ORs and 95% CIs computed among exposed male farmers using conditional logistic regression and adjusted for age, district, duration of smoking, duration of coffee drinking, MMSE, family history of PD, and use of pesticides for seeds. ^***b***^Seven missing values (one case and six controls).						

Similar analyses were performed for insecticides, fungicides, and herbicides (Table 3). For insecticides, there was a significant monotonic association with increasing quartiles of intensity only (*p*-trend = 0.04) with an OR of 2.04 (95% CI: 1.03, 4.05) for the highest versus lowest quartile. For fungicides, there was a significant positive trend for duration of exposure (*p*-trend = 0.03) with an OR of 2.28 (95% CI: 1.16, 4.50) for the highest quartile; for intensity and cumulative exposure, ORs were significantly increased for the two highest quartiles, but the association was strongest for the third quartile. No association was observed for herbicides. Interactions between duration and intensity were not statistically significant (Figure 2); for fungicides and insecticides, ORs associated with short-duration–high-intensity were > 1, whereas they were < 1 for long-duration–low-intensity.

**Table 3:** Association of PD with indicators of professional exposure to broad classes of pesticides (herbicides, fungicides, insecticides) among male farmers.

Class of pesticides	Duration of exposure			Cumulative exposure			Average exposure intensity		
	Quartile (min–max)^*a*^	OR (95% CI)^*b*^	*p*‑Trend	Quartile (min–max)^*c*^	OR (95% CI)^*b*^	*p*‑Trend	Quartile (min–max)^*d*^	OR (95% CI)^*b*^	*p*‑Trend
Herbicides	1 (< 21)	Reference		1 (< 35)	Reference		1 (< 1.95)	Reference
	2 (21–28)	0.73 (0.37, 1.45)		2 (35–74)	1.68 (0.89, 3.18)		2 (1.95–2.70)	1.24 (0.67, 2.30)
	3 (29–33)	1.01 (0.51, 1.98)		3 (75–117)	0.84 (0.41, 1.73)		3 (2.71–4.00)	0.89 (0.46, 1.73)
	4 (> 33)	1.68 (0.88, 3.22)	0.819	4 (> 117)	1.12 (0.57, 2.20)	0.931	4 (> 4.00)	1.14 (0.57, 2.26)	0.882
		AIC = 361.6			AIC = 362.8			AIC = 366.0
Fungicides	1 (< 21)	Reference		1 (< 35)	Reference		1 (< 1.50)	Reference
	2 (21–31)	0.97 (0.48, 1.96)		2 (35–98)	1.67 (0.81, 3.46)		2 (1.50–4.00)	1.78 (0.89, 3.55)
	3 (32–39)	1.83 (0.91, 3.66)		3 (99–412)	4.78 (2.16, 10.60)		3 (4.02–11.06)	3.91 (1.72, 8.89)
	4 (> 39)	2.28 (1.16, 4.50)	0.025	4 (> 412)	2.90 (1.22, 6.89)	0.063	4 (> 11.06)	2.27 (0.95, 5.44)	0.174
		AIC = 359.3			AIC = 350.8			AIC = 355.9
Insecticides	1 (< 25)	Reference		1 (< 36)	Reference		1 (< 1.04)	Reference
	2 (25–31)	0.68 (0.33, 1.38)		2 (36–55)	0.90 (0.46, 1.79)		2 (1.04–1.77)	1.58 (0.74, 3.38)
	3 (32–38)	0.99 (0.53, 1.83)		3 (56–92)	1.07 (0.58, 1.99)		3 (1.78–3.02)	1.80 (0.97, 3.35)
	4 (> 38)	1.02 (0.56, 1.85)	0.938	4 (> 92)	1.88 (0.98, 3.61)	0.075	4 (> 3.02)	2.04 (1.03, 4.05)	0.043
		AIC = 365.6			AIC = 362.4			AIC = 361.5
Abbreviations: AIC, Akaike information criterion; max, maximum; min, minimum; MMSE, Mini-Mental State Examination; PD, Parkinson’s disease. ^***a***^Expressed in number of years of exposure. ^***b***^ORs and 95% CIs computed among exposed male farmers using conditional logistic regression adjusted for age, district, duration of smoking, duration of coffee drinking, MMSE, family history of PD, and use of pesticides for seeds. ^***c***^Expressed in cumulative number of applications. ^***d***^Expressed in number of applications per year.									

Because intensity of exposure to both fungicides and insecticides was associated with PD, we also estimated their separate and joint effects (see Supplemental Material, Table S1). Compared with low-intensity exposure to fungicides and insecticides, the OR for high-intensity exposure to fungicides alone was 2.42 (95% CI: 0.99, 5.91) and the OR for high-intensity exposure to insecticides alone was 2.04 (95% CI: 0.90, 4.64). Although high-intensity exposure to both fungicides and insecticides had the highest relative risk (OR = 3.68; 95% CI: 1.61, 8.42), the joint OR was consistent with a multiplicative joint effect (*p*-interaction = 0.60).

In sensitivity analyses, adjustment for pesticide use for gardening and alternative definitions of farming led to similar results (data not shown). Intensity of pesticide exposure was associated with PD, with estimates similar to those from the main analysis, when excluding applications occurring 2, 10, or 20 years before the reference date (*p-*trend < 0.05; see Supplemental Material, Table S2). Analyses restricted to cases with disease duration ≤ 3 years confirmed our main findings (see Supplemental Material, Table S3).

**Figure 3:**
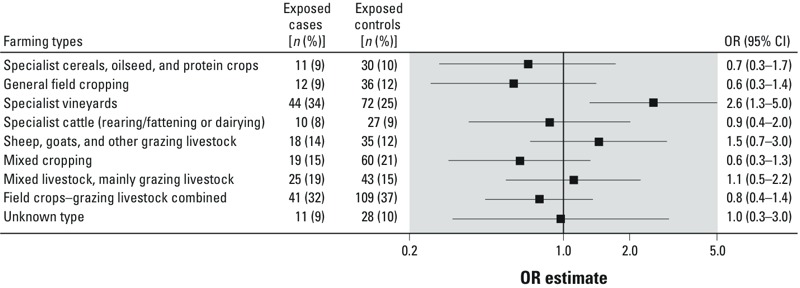
Association of PD with use of pesticides according to nine farming types among male farmers. Only farming types with &gt; 10 exposed participants are shown. ORs are adjusted for age, district, duration of smoking, duration of coffee drinking, MMSE, family history of PD, and use of pesticides for seeds. Abbreviations: MMSE, Mini-Mental State Examination; PD, Parkinson’s disease.

*PD and type of farming.* Farmers were mainly exposed to pesticides in farms defined as “field crops–grazing livestock combined” (36%), “specialist vineyards” (28%), and “mixed cropping” (19%). Compared with controls, PD cases were more frequently exposed to pesticides in farms that specialized in vineyards (34% vs. 25%; OR = 2.56; 95% CI: 1.31, 4.98; Figure 3). In these farms, intensity of use of pesticides was significantly associated with PD (for < 12 applications/year, OR = 1.79; 95% CI: 0.78, 4.09; for ≥ 12 applications/year, OR = 3.33; 95% CI: 1.57, 7.07; *p-*trend < 0.01). A similar increasing monotonic association was noted for duration (*p-*trend = 0.02) and cumulative exposure (*p*-trend < 0.01) (data not shown). There were no significant associations with other farming types (Figure 3).

*Clinical subtypes of PD and exposure to pesticides.* Among cases, 66 (50%) were classified as “tremor-dominant PD,” 59 (44%) as “non-tremor–dominant PD,” 6 (5%) as “early disease onset PD,” and 2 (1%) as “unknown subtype.” There were differences between the two most common subtypes for some clinical characteristics (main symptoms, asymmetry) but not all (age of onset, disease duration, Hoehn and Yahr stage, family history) (see Supplemental Material, Table S4).

The risk of tremor-dominant PD increased progressively with quartiles of intensity of exposure to pesticides (*p*-trend < 0.01; Table 4); the OR was 4.13 (95% CI: 1.53, 11.14) for participants in the highest quartile. For non-tremor–dominant PD, the ORs for the two highest quartiles of exposure were 2.76 (95% CI: 1.02, 7.52) and 2.26 (95% CI: 0.77, 6.64), without a significant linear trend. The test of heterogeneity for the association of the two subtypes with intensity of pesticide exposure was not statistically significant (*p* = 0.60). Among classes of pesticides, there were significant associations with intensity of insecticide exposure and fungicide exposure for tremor-dominant but not for non-tremor–dominant PD; in particular, there was a monotonic trend for intensity of insecticide exposure for tremor-dominant PD (*p-*trend < 0.01). However, both for fungicides and insecticides, the test of heterogeneity was not significant. Although the test of heterogeneity suggested a difference between tremor-dominant and non-tremor–dominant PD for herbicides, intensity of exposure to this class of pesticides was not significantly associated with any of the subtypes.

**Table 4:** Association of the main clinical subtypes of PD with intensity of exposure (average number of applications/year) among exposed male farmers.

Groups (min–max)	Controls (*n *= 298)^*a*^	Tremor-dominant PD cases (*n *= 66)^*b*^			Non-tremor–dominant PD cases (*n *= 59)			*p*‑Heterogeneity
	*n* (%)	*n* (%)	OR (95% CI)^*c*^	*p*‑Trend	*n* (%)	OR (95% CI)^*c*^	*p*‑Trend	
All pesticides
1 (0.50–3.20)	73 (25)	11 (17)	Reference		10 (17)	Reference
2 (3.22–6.01)	73 (25)	13 (20)	1.40 (0.56, 3.50)		15 (25)	1.62 (0.63, 4.17)
3 (6.04–12.51)	73 (25)	17 (26)	2.75 (1.06, 7.13)		19 (32)	2.76 (1.02, 7.52)
4 (> 12.51)	73 (25)	24 (37)	4.13 (1.53, 11.14)	0.004	15 (25)	2.26 (0.77, 6.64)	0.235	0.573
Herbicides
1 (< 2.00)	140 (47)	34 (52)	Reference		29 (50)	Reference
2 (2.06–3.39)	71 (24)	10 (15)	0.69 (0.30, 1.60)		15 (25)	1.28 (0.60, 2.77)
3 (> 3.39)	86 (29)	22 (33)	1.45 (0.71, 3.00)	0.279	15 (25)	0.68 (0.30, 1.56)	0.284	0.053
Fungicides
1 (< 2.00)	146 (50)	24 (36)	Reference		25 (42)	Reference
2 (2.06–8.73)	75 (25)	21 (32)	2.88 (1.27, 6.53)		20 (34)	1.87 (0.78, 4.46)
3 (> 8.73)	75 (25)	21 (32)	2.83 (1.11, 7.23)	0.485	14 (24)	1.67 (0.60, 4.62)	0.854	0.651
Insecticides
1 (< 1.30)	126 (42)	22 (34)	Reference		25 (42)	Reference
2 (1.31–2.65)	85 (29)	19 (29)	1.58 (0.77, 3.23)		18 (31)	1.26 (0.61, 2.62)
3 (> 2.65)	84 (28)	24 (37)	2.58 (1.23, 5.40)	0.008	16 (27)	1.08 (0.48, 2.43)	0.479	0.207
Abbreviations: MMSE, Mini-Mental State Examination; PD, Parkinson’s disease. ^***a***^Six missing values (herbicides, 1; fungicides, 2; insecticides, 3). ^***b***^One missing value for insecticides. ^***c***^ORs and 95% CIs computed among male farmers using polytomous logistic regression with controls as the reference group and adjusted for age, district, duration of smoking, duration of coffee drinking, MMSE, family history of PD, and use of pesticides for seeds.								

## Discussion

In this population-based case–control study of male farmers exposed to pesticides, fungicides and insecticides were independently associated with PD, but there was no association with herbicides. The strongest associations were observed for farmers with frequent exposures over a long period of time. These relationships remained in analyses based on exposures occurring ≥ 20 years before disease onset. In addition, farmers who applied pesticides in vineyards were at particularly increased risk. Finally, the association between PD and pesticides/insecticides was significant for tremor-dominant PD, the most frequent and typical presentation of PD.

Few PD studies have evaluated pesticide exposure quantitatively, and most of those that did mainly assessed duration and rarely cumulative exposure (Wirdefeldt et al. 2011). To our knowledge, no study has compared associations of PD with different dimensions of exposure. When we examined intensity, duration, and cumulative exposure separately, higher intensity of exposure to pesticides overall and to insecticides (i.e., more frequent applications) was the measure of exposure that displayed the strongest association with PD. For fungicides, the three characteristics of exposure were significantly associated with PD. We also found an interaction between intensity and duration of exposure to pesticides overall: The strongest association was observed for those with frequent exposures for a long period, but there was also a trend toward an association for those with frequent exposures over shorter periods. There was no association for those with infrequent exposures over long periods. Repeated exposures may lead to higher internal doses of pesticides for a longer time (Lebailly et al. 2009) and may be more toxic for dopaminergic neurons. In addition, studies conducted in France and the United States have shown that, in some settings, intensive use of pesticides is associated with more frequent preparation of pesticides before spraying and with cleaning equipment after applications—both of which lead to high exposure levels—as well as with a higher risk of acute incidents (Arbuckle et al. 2002; Lebailly et al. 2009). These findings show the value of taking into account intensity of exposure in addition to duration.

PD has a long latency period. Neuronal loss in the substantia nigra begins 3–7 years before motor symptoms appear (de la Fuente-Fernández et al. 2011). We therefore excluded exposures occurring in the 5 years before onset. Recent studies suggest that the latency period between non-motor symptoms and PD onset may be longer (Savica et al. 2010). We performed analyses with a latency period of 20 years that confirmed our main findings.

The association between PD and insecticides is consistent with previous epidemiological studies. Based on 14 studies, a meta-analysis estimated a pooled risk ratio of 1.5 for insecticides (van der Mark et al. 2012); few studies, however, have considered quantitative measures of exposure (Elbaz et al. 2009; Tanner et al. 2011). Studies based on serum or brain measures of organochlorines have shown associations of some compounds (dieldrin, hexachlorocyclohexane) with PD (Richardson et al. 2011; Weisskopf et al. 2010). In addition, *in vivo* and *in vitro* studies show the ability of some insecticides (e.g., dieldrin, rotenone) to reproduce some PD features (e.g., neuronal loss, decreased motor activity) or to induce cellular mechanisms observed in PD (e.g., oxidative stress, protein aggregation) (Hatcher et al. 2008).

A recent meta-analysis showed no association between PD and fungicides (OR = 0.99; 95% CI: 0.71, 1.40), but this finding was based on nine studies only (van der Mark et al. 2012). In the present study, the association with fungicides did not seem to be explained by their correlation with insecticides because both exposures appeared to have independent effects. French agriculture is characterized by one of the highest levels of fungicide use (Food and Agriculture Organization of the United Nations 2012). Interestingly, a study performed in California showed an association of ambient exposure to carbamate fungicides (maneb, ziram) and PD (Wang et al. 2011). Thus, fungicides may have a true effect; further epidemiological and toxicological studies are needed.

Exposure assessment represents one of the main difficulties in epidemiological studies of pesticides, where findings can be affected by recall bias, in particular when detailed exposure data are elicited (Rugbjerg et al. 2011). An alternative way of studying the role of pesticides involves examining their target (i.e., in which context pesticides are used) because there are marked differences in patterns of pesticides use across types of faming (Farm Accountancy Data Network 2009b). We found an association of PD with use of pesticides in vineyards. This association likely reflects the high level of pesticides used on these farms (European Commission 2000); for instance, vineyards rank second regarding insecticide use and first regarding fungicide use. In the present study, the median number of pesticide applications per year among exposed farmers working in vineyards was 12.3. Farms specialized in orchards rank first in terms of insecticides use, but only eight participants (two cases, six controls) were exposed to pesticides from this type of farming in our study.

To our knowledge, only one study has compared clinical features of PD patients occupationally exposed to pesticides with those of unexposed patients, but without considering PD subtypes, and reported no major differences (Tanner et al. 2011). However, only specific pesticides (previously associated with mitochondrial complex I inhibition or oxidative stress) were investigated, therefore leading to few exposed participants. In our study, although differences between tremor-dominant and non-tremor–dominant PD were not statistically significant, insecticides were positively associated with tremor-dominant PD, whereas there was no evidence of an association with non-tremor–dominant PD. This may be interpreted in two complementary ways. First, rest tremor in PD can be easily identified and has high diagnostic specificity because it is less frequent in other causes of parkinsonism (Suchowersky et al. 2006). It is possible that diagnostic misclassification may be more common among non-tremor–dominant cases due to the less specific nature of symptoms, therefore biasing association estimates toward the null. However, substantial misclassification is unlikely in the present study given that all diagnoses were confirmed by neurologists with experience in movement disorders. Thus, although we cannot rule out some bias due to outcome misclassification, it is unlikely to fully explain our findings. Second, as suggested by some authors (de la Fuente-Fernández et al. 2011; Marras and Lang 2013; Obeso et al. 2010), different pathophysiological mechanisms may be involved in different clinical subtypes. One study found that non-tremor–dominant cases were more likely to have neocortical Lewy body disease, whereas tremor-dominant cases were more likely to have brainstem/limbic pathology (Marras and Lang 2013; Selikhova et al. 2009). Insecticides and fungicides may be more particularly associated with tremor-dominant PD because of the vulnerability of dopaminergic neurons in the substantia nigra to mitonchondrial inhibitors and oxidative stressors; further work is needed to better understand the etiology of PD subtypes. Nevertheless, our findings show that, whatever the explanation for the stronger association of pesticides with tremor-dominant PD, insecticides and fungicides were associated with the most typical form of PD.

Our findings need to be considered in light of some limitations. First, the retrospective collection of exposure data represents the main limitation of this study. However, pesticide data were collected by eliciting information in a standardized sequential manner, from less to more detailed, in order to help participants report exposures. We requested information only on classes of pesticides rather than on specific chemical products, but ancillary information obtained on chemical products was used to assess the likelihood of the reported data. All questionnaires were carefully discussed, and additional information was collected in a sizeable number of instances. Analyses were adjusted for a measure of cognitive performance to take into account potential differences in recall related to cognitive status; in addition, analyses restricted to cases with short-disease duration, and therefore more likely to be cognitively intact, were consistent with our main findings. Second, we did not include information about use of personal protective equipment (PPE). The impact of PPE use on our results is likely to be limited because PPE were rarely used by French farmers during the periods when most participants worked (Lebailly et al. 2009). In addition, the protective effect of PPE is debated: It has been suggested that PPE may represent a source of secondary exposure to pesticides (Hines et al. 2001), and the efficiency of some PPEs is questioned (Anses 2010). Finally, all analyses were restricted to men, and our findings are not generalizable to women who have very different patterns of exposure to pesticides.

Strengths of the study include its population-based design, the confirmation of PD diagnoses by a neurologist, and a comprehensive collection of pesticide exposure data allowing us to compare different measures of pesticide exposure. Acceptance rates were high and comparable among male cases and controls. Note that the acceptance rate was computed among male participants overall and was not specific to the male exposed farmers included in the analyses. Moreover, we examined the association between PD and exposure to pesticides while taking into account the clinical heterogeneity of the disease using previously defined common clinical subtypes.

## Conclusions

This study helps to better characterize pesticide exposures associated with a higher risk of PD, and it demonstrates the value of defining clinical phenotypes in order to understand underlying mechanisms.

## Supplemental Material

(510 KB) PDF
