# Supplementary material for: Association of Parkinson’s Disease and Its Subtypes with Agricultural Pesticide Exposures in Men: A Case–Control Study in France
Source: Environ Health Perspect. 2015 Mar 27;123(11):1123–9. doi: 10.1289/ehp.1307970 (PMC4629732; doi:10.1289/ehp.1307970)
Supplement: (510 KB) PDF [file ehp.1307970.s001.acco.pdf]

Environ Health Perspect
doi: 10.1289/ehp.1307970

Note to Readers: ***EHP*** **strives to ensure that all journal content is accessible to all readers. However, some figures and Supplemental Material published in *EHP* articles may not conform to 508 standards due to the complexity of the information being presented. If you need assistance accessing journal content, please contact ehp508@niehs.nih.gov. Our staff will work with you to assess and meet your accessibility needs within 3 working days.**

Supplemental Material

Association of Parkinson's Disease and Its Subtypes with Agricultural Pesticide Exposures in Men: A Case–Control Study in France

Frédéric Moisan, Johan Spinosi, Laurène Delabre, Véronique Gourlet, Jean-Louis Mazurie, Isabelle Bénatru, Marcel Goldberg, Marc G. Weisskopf, Ellen Imbernon, Christophe Tzourio, and Alexis Elbaz

Table of Contents

Figure S1. Indicators of pesticide exposure. [a]Reference date: for cases, the year of PD onset; for controls, the year of PD onset of the matched case.

Table S1. Independent and joint effects of intensity of exposure to insecticides and fungicides among male farmers.

Table S2. Association of Parkinson's disease with indicators of professional exposure to pesticides among male farmers according to three latency periods.

Table S3. Association of Parkinson's disease with indicators of professional exposure to broad classes of pesticides (herbicides, fungicides, insecticides) among male farmers restricted to cases with disease duration of 3 years or less.

Table S4. Characteristics of controls and tremor dominant and non-tremor dominant Parkinson's disease cases among male farmers.

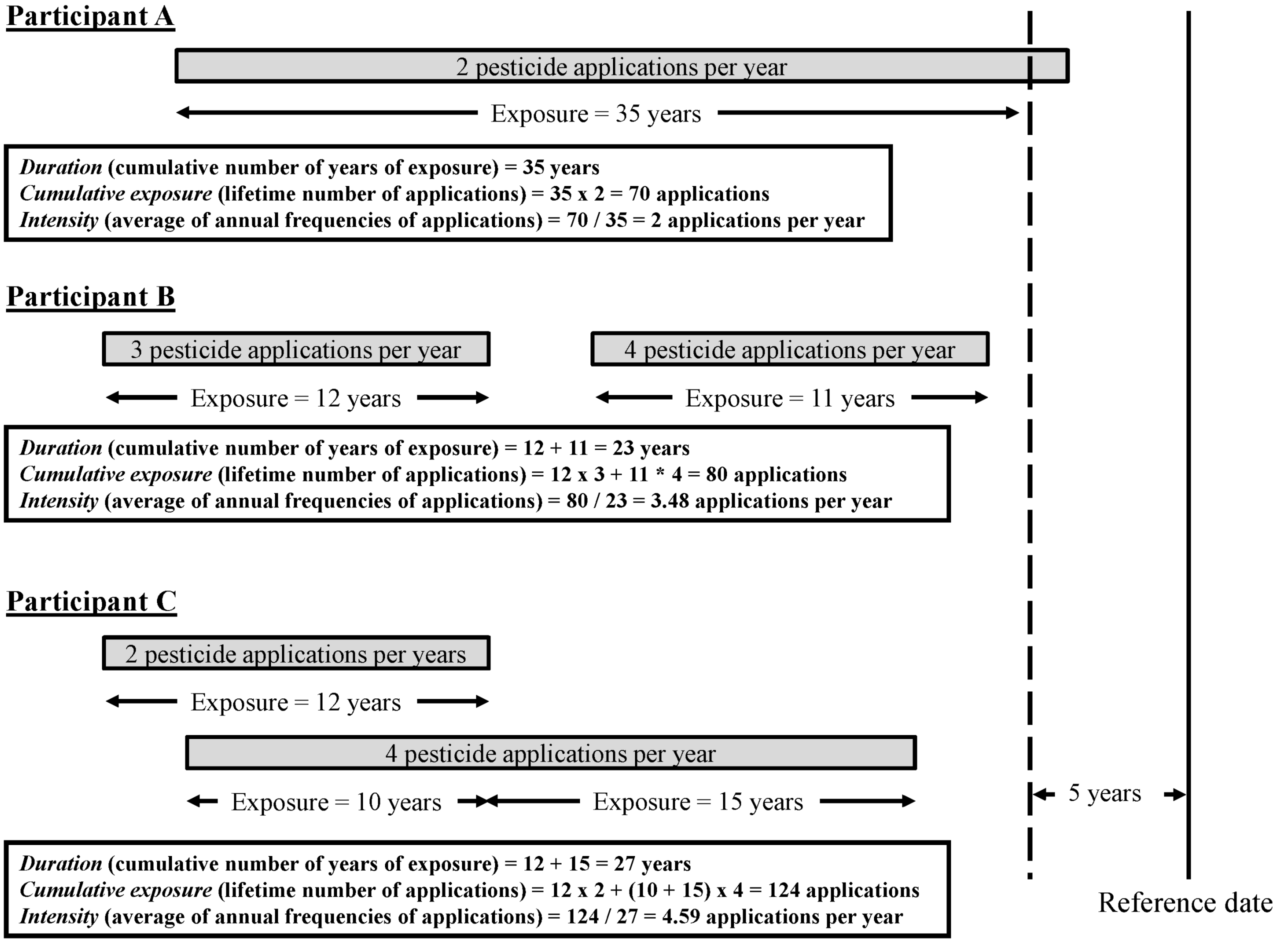

Figure S1. Indicators of pesticide exposure. [a]Reference date: for cases, the year of PD onset; for controls, the year of PD onset of the matched case.

Table S1. Independent and joint effects of intensity of exposure to insecticides and fungicides among male farmers.

Intensity of fungicide exposure[a]	Intensity of insecticide exposure[a]	Cases (*n* = 133)[b] *n* (%)	Controls (*n* = 298)[b] *n* (%)	OR (95% CI)[c]
Low[d]	Low	16 (12)	64 (22)	Reference
High[e]	Low	26 (20)	48 (16)	2.42 (0.99, 5.91)
Low	High	25 (19)	61 (21)	2.04 (0.90, 4.64)
High	High	65 (49)	120 (41)	3.68 (1.61, 8.42)

[a]Expressed in average number of applications per year. [b]Six missing values (one case and five controls). [c]Odds ratios and 95% confidence intervals (OR, 95% CI) were computed among male farmers using conditional logistic regression and adjusted for age, district, duration of smoking, duration of coffee drinking, MMSE, family history of PD, and use of pesticides for seeds. [d]Q1 (not exposed or exposure <1.04 applications per year for insecticides and <1.50 applications per year for fungicides). [e]Q2 (1.04-1.77 for insecticides, 1.50-4.00 for fungicides) + Q3 (1.78-3.02 for insecticides, 4.02-11.06 for fungicides) + Q4 (>3.02 for insecticides, >11.06 for fungicides).

Table S2. Association of Parkinson's disease with indicators of professional exposure to pesticides among male farmers according to three latency periods.

Latency period/Groups	Duration of exposure OR (95% CI)[a]	*p* for trend	Cumulative exposure OR (95% CI)[a]	*p* for trend	Average exposure intensity OR (95% CI)[a]	*p* for trend
Two years[b]						
1	Reference		Reference		Reference	
2	1.20 (0.62, 2.33)		0.86 (0.42, 1.74)		1.18 (0.59, 2.37)	
3	1.22 (0.63, 2.35)		1.57 (0.81, 3.08)		2.08 (1.00, 4.33)	
4	3.27 (1.41, 7.59)	0.384	1.80 (0.85, 3.80)	0.089	2.69 (1.21, 5.98)	0.014
Ten years[b]						
1	Reference		Reference		Reference	
2	0.70 (0.35, 1.39)		0.71 (0.34, 1.48)		1.10 (0.56, 2.17)	
3	1.07 (0.55, 2.09)		1.23 (0.61, 2.47)		1.61 (0.78, 3.35)	
4	1.20 (0.60, 2.38)	0.515	1.99 (0.95, 4.21)	0.018	2.83 (1.27, 6.28)	0.007
Twenty years[b,c]						
1	Reference		Reference		Reference	
2	0.73 (0.39, 1.36)		0.50 (0.23, 1.09)		0.68 (0.33, 1.40)	
3	1.13 (0.55, 2.33)		1.34 (0.68, 2.61)		1.67 (0.83, 3.37)	
4	1.17 (0.58, 2.36)	0.364	1.63 (0.77, 3.49)	0.072	2.20 (1.02, 4.75)	0.021

Abbreviations: CI, confidence interval; MMSE, mini-mental state examination; OR, odds ratio; PD, Parkinson's disease.

[a]Odds ratios and 95% confidence intervals were computed among male farmers using conditional logistic regression adjusted for age, district, duration of smoking, duration of coffee drinking, MMSE, family history of PD, and use of pesticides for seeds. [b]Seven missing values (one case, six controls). [c]Analyses conducted among 132 exposed cases and 293 exposed controls (one case and five controls were not considered exposed when the last 20 years were not taken into account).

Table S3. Association of Parkinson's disease with indicators of professional exposure to broad classes of pesticides (herbicides, fungicides, insecticides) among male farmers restricted to cases with disease duration of 3 years or less.

Classes of pesticides/Groups	Ca/Co *n*	Duration of exposure OR (95% CI)[a]	Ca/Co *n*	Cumulative exposure OR (95% CI)[a]	Ca/Co *n*	Average exposure intensity OR (95% CI)[a]
Herbicides						
Low[b]	8/33	Reference	9/27	Reference	10/28	Reference
High[c]	26/49	3.19 (1.19, 8.51)[d]	25/55	1.91 (0.71, 5.15)[d]	24/54	1.73 (0.62, 4.85)[d]
Fungicides						
Low	9/43	Reference	6/35	Reference	8/35	Reference
High	25/39	5.90 (1.70, 20.44)[e]	28/47	6.49 (1.68, 25.00)[e]	26/47	3.10 (1.02, 9.46)[e]
Insecticides						
Low	9/32	Reference	8/31	Reference	8/36	Reference
High	25/50	2.24 (0.89, 5.77)[f]	26/51	2.75 (0.94, 8.06)[f]	26/46	5.20 (1.59, 17.00)[f]

Abbreviations: Ca, cases; CI, confidence interval; Co, controls; MMSE, mini-mental state examination; OR, odds ratio; PD, Parkinson's disease. [a]Odds ratios and 95% confidence intervals computed among exposed male farmers using conditional logistic regression adjusted for age, district, duration of smoking, duration of coffee drinking, MMSE, family history of PD, and use of pesticides for seeds. [b]Unexposed or in the first quartile of exposure (See Table 3 for cut-off). [c]In the second, third or fourth quartile of exposure (See Table 3 for cut-off). [d]Corresponding estimates including all cases and controls were 1.09 (0.65, 1.82) for duration, 1.23 (0.73, 2.09) for cumulative exposure, and 1.09 (0.66, 1.81) for average exposure intensity. [e]Corresponding estimates including all cases and controls were 1.56 (0.91, 2.66) for duration, 2.51 (1.35, 4.67) for cumulative exposure, and 2.17 (1.17, 4.05) for average exposure intensity. [f]Corresponding estimates including all cases and controls were 0.91 (0.57-1.47) for duration, 1.23 (0.76, 2.00) for cumulative exposure, and 1.83 (1.08, 3.09) for average exposure intensity.

Table S4. Characteristics of controls and tremor dominant and non-tremor dominant Parkinson's disease cases among male farmers.

Characteristic	Tremor dominant cases	Non-tremor predominant cases	Controls
n	66	59	298
Age of onset (years), median	67.5	68.0	n.a.
Duration of PD (years), median	6	6	n.a.
Main signs at disease onset, *n* (%)[a]			
Bradykinesia	14 (21)	50 (85)	n.a.
Rigidity	11 (17)	36 (62)	n.a.
Tremor	62 (94)	16 (27)	n.a.
Asymmetry of symptoms at disease onset, *n* (%)	59 (89)	44 (74)	n.a.
Hoehn and Yahr scale, median	2.5	2.5	n.a.
Smoking			
Ever smoking, *n* (%)	27 (41)	14 (24)	154 (52)
Duration of smoking[b], median	18	27	30
Coffee drinking			
Ever consumed, *n* (%)	55 (83)	42 (74)	250 (84)
Duration of coffee drinking[c], median	50	50	54
MMSE, median	26	24	27
Family history of PD (parents, siblings), *n* (%)	6 (9)	5 (9)	10 (3)
Parents (at least one) worked as farmers, *n* (%)	63 (96)	55 (98)	279 (95)

Abbreviations: MMSE, mini-mental state examination; n.a., not applicable; PD, Parkinson's disease.

[a]Sum exceeds 100% since cases can have multiple signs at disease onset. [b]Among ever smokers. [c]Among ever coffee drinkers.
